# Early life adversity, reproductive history and breast cancer risk

**DOI:** 10.1093/emph/eoac034

**Published:** 2022-08-23

**Authors:** Amy M Boddy, Shawn Rupp, Zhe Yu, Heidi Hanson, Athena Aktipis, Ken Smith

**Affiliations:** Department of Anthropology, University of California Santa Barbara, Santa Barbara, CA, USA; Biodesign Center for Biocomputing, Security, and Society, Arizona State University, Tempe, AZ, USA; Population Sciences, Huntsman Cancer Institute, University of Utah, Salt Lake City, UT, USA; Department of Surgery, University of Utah, Salt Lake City, UT, USA; Department of Sociology, University of Utah, Salt Lake City, UT, USA; Department of Psychology & Center for Evolution and Medicine, Arizona State University, Tempe, AZ, USA; Department of Family and Consumer Studies and Population Science/Huntsman Cancer Institute, University of Utah, Salt Lake City, UT, USA

**Keywords:** early life adversity, breast cancer, health disparity, life history, reproduction

## Abstract

**Background and objectives:**

Individuals who experience early life adversity are at an increased risk for chronic disease later in life. Less is known about how early life factors are associated with cancer susceptibility. Here, we use a life history framework to test whether early life adversity increases the risk of breast cancer. We predict that early life adversity can shift investment in somatic maintenance and accelerate the timing of reproduction, which may mediate or interact with the risk of breast cancer.

**Methodology:**

We use population-wide data from the Utah Population Database (UPDB) and Utah Cancer Registry, leading to 24 957 cases of women diagnosed with breast cancer spanning 20 years (1990–2010) and 124 785 age-matched controls. We generated a cumulative early life adversity summation score to evaluate the interaction (moderation) and mediation between early life adversity, reproductive history and their association with breast cancer risk.

**Results:**

Our analyses led to three key findings: (i) more early life adversity, when considered as a main effect, accelerates the time to first birth and death, (ii) early age at first birth and high parity decreases the risk of breast cancer and (iii) we find no association between early adversity and breast cancer risk either as a main effect or in its interaction with reproductive history.

**Conclusion and implications:**

Early adversity elevates the risk of overall mortality through mechanisms other than breast cancer risk. This suggests early life factors can generate different effects on health. Future work should incorporate more complex view of life history patterns, including multiple life stages, when making predictions about cancer susceptibility.

## INTRODUCTION

Individuals with multiple adverse early life events are at an increased risk for poor late-onset health outcomes, including inactivity, obesity, diabetes, cancer, heart disease, mental ill health and problematic alcohol or drug use [1–3]. Adverse early life events include, abuse, neglect, household challenges, such as parental death or illness, parental separation or divorce, parental incarceration, domestic violence, and family poverty [2]. Many of these adverse childhood events, such as unstable family environments, divorce and poor social networks, can result in behavioral problems and chronic health conditions later in life [2]. There is accumulating support that there are biological mechanisms beyond health harming behaviors (e.g. substance abuse, smoking, physical inactivity), linking early life adversity and chronic diseases such as cancer [4–6].

Currently, there are multiple theoretical frameworks for understanding this link between adverse conditions early in life and adult health and survival, including allostatic load [7, 8] and adaptive calibration models [9] in the psychology literature, transition to adulthood and life course perspectives in sociology, demography and epidemiology [10], and predictive adaptive response models [11, 12]. Here, we apply principles from evolution and ecology theory to understand the links between early life challenges and disease risk later in life. Life history theory postulates that organisms encounter trade-offs between the allocation of resources toward reproduction and survival [13]. Environmental conditions affect both selection for life history strategies (e.g. timing of first birth) and calibration of life history strategies to meet the challenges and demands of the environment. Applying these life history principles to human health, individuals that experience adverse conditions early in life may shift investment into reproduction over somatic maintenance. This reduction in somatic maintenance may provide one explanation for the link between early adversity and chronic disease later in life, including cancer susceptibility.

To test these predictions, we focus on breast cancer, the most commonly diagnosed cancer in women in the USA and globally [14] and the second leading cause of cancer-related deaths [14]. Risk factors for breast cancer can be contradictory, suggesting that multiple mechanisms underlie this risk and that they may interact in complex ways to shape cancer risk. Breast cancer is often characterized as a disease of reproductive mismatch, whereas late age at first birth (AFB) and low parity are risk factors [15–20]. While less is known about how early life factors may be associated with later life breast cancer susceptibility, several studies have linked stressful early-life events to breast cancer diagnoses later in life [6, 21]. This includes stressful life events during early development [22] and maternal death during childhood [23]. Yet, other studies fail to show the link between early stress and breast cancer risk [24, 25]. The lack of an association in the later studies might be due to the complexity of breast cancer risk factors, including the mediating effects of reproductive history.

The accumulation of early adverse life events may set individuals on a distinct life history trajectory leading to more morbidity and mortality later in life [26]. Exposure to stressful and unstable environments have been shown to accelerate reproductive maturity and reproductive aging, including lower menarcheal age, earlier AFB and high parity [27–29]. This may come at the expense of somatic investment (due to reduced immune function or cell cycle control), leading to greater susceptibility to cancer. However, when considering breast cancer risk, previous work demonstrates investment in reproduction, such as early AFB and high parity [15–17, 19], decreases an individual’s risk for breast cancer. This suggests there may be underlying tensions among breast cancer risk factors, making it difficult to identify a singular role of life history factors such as fertility timing and early adversity in shaping disease risk.

Here, we investigate the roles of early life adversity and accelerated reproduction in shaping breast cancer risk. We examine whether individuals who experience high childhood adversity have an increased risk for breast cancer and then we examine whether there is mediation and effect moderation (interaction) between adversity and reproductive factors and breast cancer risk. We predict that high early life adversity is associated with the risk for breast cancer, beyond the increase in risk that comes from lower fertility and delayed reproduction. In other words, adversity and lower/delayed fertility should have synergistic effects on breast cancer risk. Higher fertility and earlier reproduction would be expected to decrease the risk of breast cancer in individuals who experienced greater early adversity.

To investigate the association between cumulative early life adversity and breast cancer risk, we leveraged linked data from the Utah Population Database (UPDB) and the Utah Cancer Registry (UCR), which have extensive demographic and medical information on individuals across multiple generations. To test for the effects of early adversity, we generated a novel cumulative adversity score (CAS), using demographic and administrative data. This is, to our knowledge, the first study to look at the interaction between early adversity measures and reproductive variables in breast cancer risk.

## METHODS

### Data

This study utilizes data drawn from the UPDB. The UPDB is one of the world’s richest sources of linked population-based information for demographic, genetic and epidemiological studies. UPDB has supported numerous biomedical investigations in large part because of its size, inclusion of multi-generational pedigrees and linkages to numerous data sources. The UPDB contains data on over 11 million individuals from the late 18th century to the present. UPDB data represent Utah’s population that appears in administrative and historical records. The holdings of the data grow due to longstanding efforts to update records as they become available including statewide birth and death certificates, hospitalizations, ambulatory surgeries and driver licenses. UPDB creates and maintains links between the database and the medical records held by the two largest healthcare providers in Utah as well as Medicare claims. The multigenerational pedigrees representing Utah’s founders and their descendants were constructed based on data provided by the Genealogical Society of Utah (GSU). These pedigrees spanning the past century have been expanded extensively based on vital records and, together with the GSU data, form the basis of the deep genealogical structure of the UPDB. The overall structure of the UPDB provides the basis for identifying events and conditions that span a person’s lifetime including births and deaths among kin as well as family structure and socioeconomic status. The UPDB comprehensively links statewide cancer incidence data from the UCR, a National Cancer Institute supported Surveillance, Epidemiology, and End Result (SEER) registry that collects data on all incident cancers in Utah except non-melanoma skin cancer. The UCR started in 1966 and became a SEER registry in 1973. This study has been approved by the University of Utah’s Resource for Genetic and Epidemiologic Research and its Institutional Review Board. The UPDB has been used for a wide range of population-based studies [30]. To access cancer diagnosis, we relied on these existing links between the UPDB and the UCR. Using the years, we identified primary breast cancer diagnoses cases, 1990–2010, and age-matched women for a 1:5 case–control ratio.

### Cumulative adversity score

We developed a new measure designed to capture stressors across the life course that may affect later life health. The CAS is based on six sociodemographic measures (Table 1) including low social-economic status (SES), parental/sib death during childhood, born to a teen mother and a low SES: high children ratio. The CAS measure is designed using the same theoretical framework as the Adverse Childhood Experience (ACE) measures; however, it is constructed using events recorded on administrative records. We leveraged the multigenerational demographic data in UPDB to construct a longitudinal record of early life conditions of all individuals, referred to as egos.

**Table 1.** Descriptive statistics for early life variables used to estimate cumulative adversity score

	Controls (*n* = 72 022)	Cases (*n* = 14 859)	Overall (*n* = 86 881)
Cumulative adversity score			
Mean (SD)	0.718 (0.931)	0.675 (0.909)	0.711 (0.927)
Median [Min, Max]	0 [0, 5.00]	0 [0, 5.00]	0 [0, 5.00]
Early death of mother	1388 (1.9%)	308 (2.1%)	1696 (2.0%)
Early death of father	2333 (3.2%)	460 (3.1%)	2793 (3.2%)
Born to teen mother	2300 (3.2%)	450 (3.0%)	2750 (3.2%)
Low SES	18 596 (25.8%)	3642 (24.5%)	22 238 (25.6%)
Number of siblings			
Mean (SD)	5.22 (2.75)	4.97 (2.73)	5.18 (2.75)
Median [Min, Max]	5 [1, 28]	5 [1, 20]	5 [1, 20]
Sibling death	18 023 (25.0)	3428 (23.1%)	21 451 (24.7%)
Low SES: high children	28 049 (38.9%)	5359 (36.1%)	33 408 (38.5%)

#### Parent’s age at the time of birth

Parent’s age at ego birth was determined by using the ego’s birth year. An indicator variable was created to flag whether the mother was 18 years or younger at the time of ego’s birth.

#### Childhood SES

The patient’s childhood socio-economic status (SES) was determined using both parents’ Socio-Economic Index (SEI) and Nam Powers scores. The higher of either parents’ SEI and Nam Power’s scores were recorded for the ego.

#### Death of a sibling

We recorded the number of siblings known to have died when ego was <18 years old.

#### Death of parent during childhood

We recorded whether the parent died before the ego was 10 years old, and whether the parent was still alive when the ego turned 18. We determined whether the parent was still alive when the ego was diagnosed using the parent’s last living date and the ego’s date of diagnosis.

#### Cumulative adversity score

We then developed a CAS to summarize the early life adversity for each individual. We assigned a point for each adverse event. This approach used the presence or absence of adverse events. If data were missing with respect to a specific component of CAS, we assumed it to be absent for that particular adverse event. These events were: (i) ego’s mother died before ego was 10 years old, (ii) ego’s father died before ego was 10 years old, (iii) ego was born to young mother (<18 years old), (iv) any sibling death, (v) ego’s socio-economic status was in bottom 25% of all records and (vi) if ego resided in a household with more than five siblings *and* was in the lowest SES quartile. We summed all points together to create the CAS. Finally, we mean-centered CAS in order to address collinearity concerns when CAS is used in an interaction with reproductive variables.

#### Reproductive history

Categorical variables were used to capture reproductive history. AFB was classified in a manner similar to previously published cut-offs [31], which include (i) nulliparous, (ii) <20 years, (iii) between 20 and 24 years (which serves as the reference group in the statistical analyses), (iv) between 24 and 29 years and (v) >30 years [32]. We coded parity into comparable bins: (i) nulliparous, (ii) ≤2 children, (iii) between 2 and 5 children (which serves as the reference group in the statistical analyses) and (iv) >5 children.

### Sample characteristics

**Figure 1.**
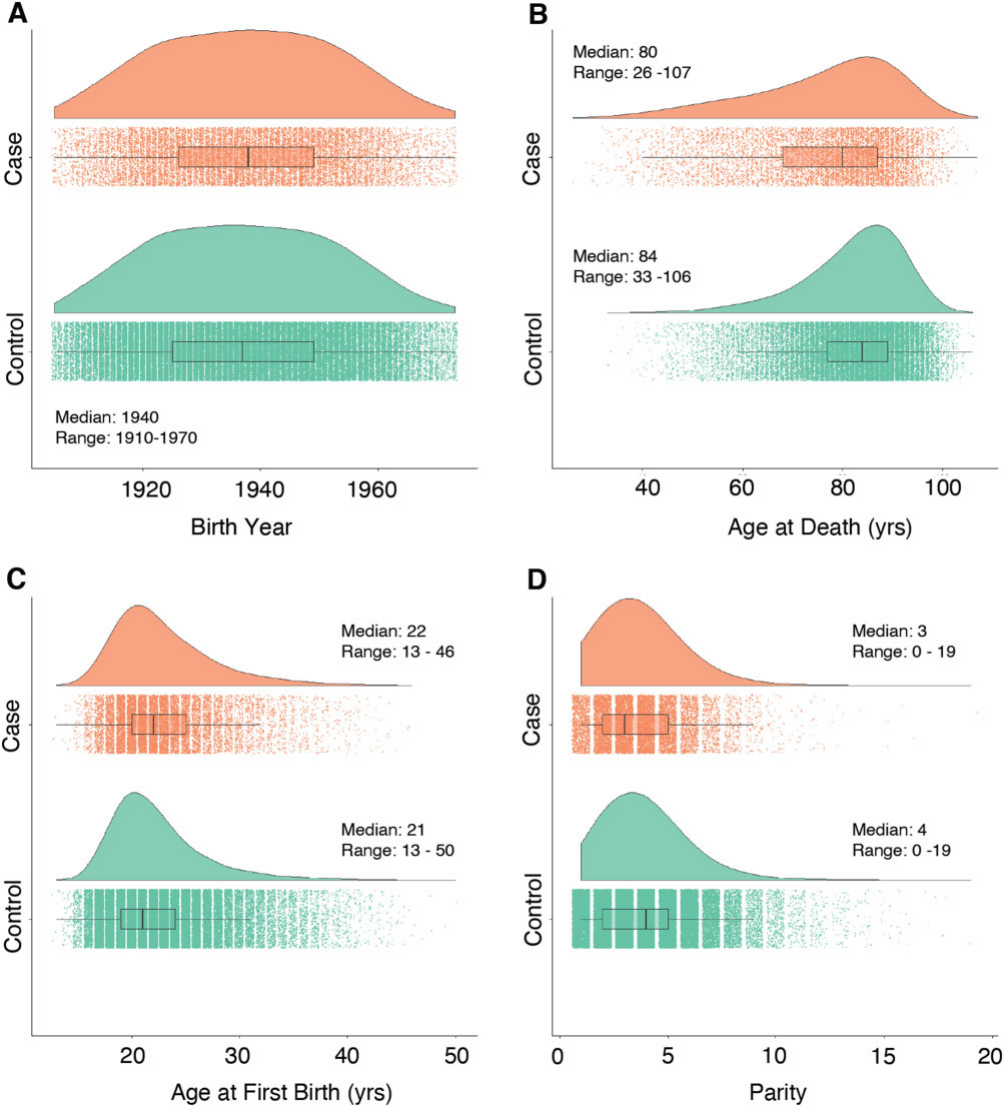
Life course summary statistics for women in the UPDB, a large multigenerational database of individuals living in Utah, include (**A**) birth year, (**B**) age at death, (**C**) age at first birth and (**D**) parity. Data represent 14 859 cases of individuals diagnosed with breast cancer and 72 022 controls. Yrs, years

To assess the relationship between early life adversity and breast cancer risk, we used data from UPDB, which allowed us to identify 24 957 women diagnosed with breast cancer and 124 785 age-matched controls. In order to observe the full period of fecundity, we only included women who survived to at least age 45 in this study. After imposing data eligibility restrictions, we report a total of 86 881 individuals in the dataset, with 14 859 breast cancer cases and 72 022 controls. After filtering, birth years ranged from 1910 to 1970 and the median age of death was 80 years (Fig. 1). Median AFB for both cases and controls were 22 years (range: 13–50 years) with median parity of four children (range: 1–19 children). For the individuals diagnosed with breast cancer, the average age of diagnosis was 65 years. The majority of individuals in the dataset scored 0 on CAS, with cases having a slightly lower adversity score than controls (Table 1 and Supplementary Fig. S1).

### Statistical methods

Cox proportional hazards models were used first to estimate the association between CAS and all-cause mortality [33, 34]. The censoring variable indicates whether there was a death (no censoring) or whether the individual was last seen alive (right censored). This model represents a robustness check for our novel CAS measures. All Cox models control for age of the individual in 1990. This date is significant because it is the time in which we pulled the breast cancer data from the UCR dataset. Death of a sibling during childhood is considered an adversity measure in our CAS score, therefore, we controlled for number of siblings to account for differences in the possibility of sibling death. Cox models were run using the survival package in R [35, 36]. Next, we estimated the relationship between reproductive history and breast cancer diagnosis (outcome variable) using a nested generalized linear models (GLM) [37] in R [36]. We tested two categorical variables of reproductive history: (i) AFB and (ii) parity (see section *Reproductive history* above for more details). We estimated the relationship between the CAS, reproductive history and whether there was a breast cancer diagnoses (i.e. cases vs controls). We used nested GLM models to fully explore the relationship between CAS and reproductive history in two distinct ways: (i) complete dataset (including nulliparous women; *n* = 86 881) and (ii) parous dataset (excludes nulliparous women; *n* = 73 727). Results based on the parous subset are published in Supplementary Materials. We analyzed the association between breast cancer diagnosis and AFB and then parity in separate GLM models. We used separate models to isolate the effects of each in recognition of their strong collinearity. Finally, interaction models were estimated where we included a multiplicative interaction term, adversity score × reproductive variable (AFB or parity) to test whether these measures, when considered jointly, additionally influence breast cancer diagnosis beyond their main effects. All nested GLM and interaction models were estimated in R [36] and adjusted for birth year and number of siblings. Odds ratios were estimated from the jtools package [38] in R.

## RESULTS

Women in this dataset were born between 1910 and 1970, with ages ranging from 26 to 107 years (Fig. 1). There was a large distribution of reproductive variables in this dataset, with the AFB ranging from 13 to 50 years and the number of children ranging from 0 to 19 children. We then generated the CAS measure using the six variables related to the individual’s early life (Table 1). The CAS score ranged from 0, which indicated the individual had no recognized adversity based on our measures, to a cumulative score of 5, indicating the individual experienced five of the six measured adverse events. The mean CAS was 0.711 (SD = 0.927) for the full dataset and no individuals had all six measures of adversity (Supplementary Fig. S1).

We estimated a Cox regression model where we find a positive relationship between CAS and the hazard rate for all-cause mortality (Cox model *N* = 86 881; HR = 1.05, *P* < 0.001 (Supplementary Table S2)). Next, we test the relationship between CAS on, in succession, AFB and parity. We find high adversity is associated with earlier ages of first birth (*P* < 0.001), but that it is not associated with parity (Supplementary Table S3).

Our analysis confirms nulliparity and late AFB are associated with an increased risk of breast cancer, while early AFB is associated with a reduced risk (Supplementary Fig. S3 and Table S4), as consistently reported in the breast cancer literature [15, 16, 18, 19]. We also found that low parity (*n* ≤ 2 children) is associated with an increased risk of breast cancer (Supplementary Fig. S3 and Table S4). Due to some data availability issues relating to verified nulliparity (i.e. where women with no observed children include both true nulliparous women as well as those for whom their reproductive history is missing), we also estimated models based on a strictly parous dataset. Results based on these parous women do not substantively alter the interpretation of results reported in the full dataset (Supplementary Table S5).

**Figure 2.**
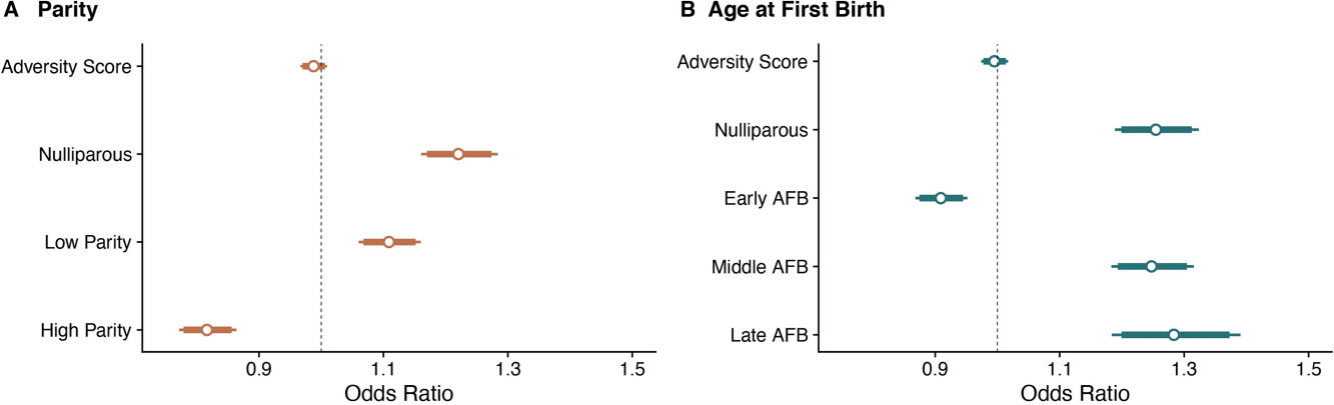
Effects of CAS and reproductive history on breast cancer diagnosis. (**A**) Females with high parity (>2 children) had a decreased risk of developing breast cancer compared to nulliparous or low parity (<2 children) individuals and (**B**) females with an early age at first birth (<20 years) had a decrease risk of developing breast cancer compared to nulliparous females and females who gave birth to first child over the age of 24 years. CAS had no significant effect on breast cancer. All generalized linear models controlled for birth year and number of siblings. Odds ratio was estimated from jtools [38] in R [36]

**Figure 3.**
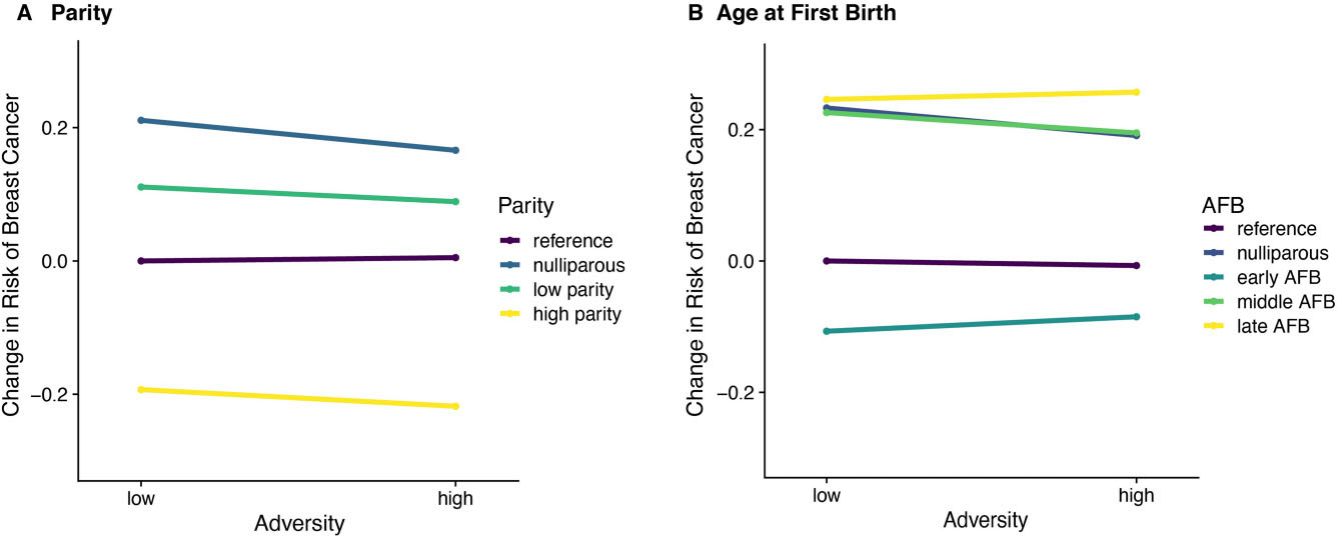
Interaction between CAS and reproductive history on breast cancer. (**A**) Interaction between CAS and parity show no significant effects on breast cancer risk. Reference group is compared to itself (2–5 children), nulliparous group = 0 children, low parity group <2 children and high parity group >5 children. (**B**) Interaction between CAS and age at first birth show no significant effects on breast cancer risk. Reference group compared to itself (20–24 years), nulliparous group = 0 children, low AFB group <20 years, middle AFB group = 24–29 years and late AFB group >30 years. *Y*-axis represents the change in risk of breast cancer associated with each combination of variables relative to the baseline: CAS = 0 and parity = 2–5 children in (A) and AFB = 20–24 years in (B). *X*-axis represents one unit change in CAS. All models were adjusted for birth year and number of siblings. AFB, age at first birth

We find CAS has no association with the risk of breast cancer. Specifically, higher adversity is not associated with breast cancer risk (Fig. 2, Supplementary Table S6). We then consider whether CAS interacts with parity and AFB and find no significant interaction effects that influence breast cancer risk (Fig. 3, Supplementary Table S8).

Low parity is a well-established risk factor for breast cancer and we found that, for low parity women, their risk for breast cancer declines as CAS increases. For nulliparous women, where the risks are greatest overall, the interaction with CAS is minimal. These results were unexpected given that higher CAS leads to higher mortality. We therefore ran an ancillary analysis on adversity score and age of diagnosis, rather than the presence or absence of breast cancer. We found that women with lower adversity scores have an earlier age of diagnosis (Supplementary Fig. S4).

## DISCUSSION

We created a novel CAS for early life events to investigate the roles of reproductive factors and early adversity in shaping breast cancer risk. We found an association between greater early adversity and mortality. Considerable work has shown that individuals who experience adverse childhood events, such as low socioeconomic conditions, have lower overall survival [39]. Here, we extend this line of work, showing a relationship between cumulative early life adversity and timing of first birth, an important life history measure. Individuals who experienced high childhood adversity have significantly earlier ages at first birth, highlighting the value of the life history framework for studying the link between adversity and reproductive schedules [11, 27].

It is known that individuals who experience low socioeconomic conditions in childhood have an increased risk for developing chronic diseases, such as cardiovascular disease [40] and diabetes [41] later in life. Here, we were able to look specifically at breast cancer incidence in relation to early adversity. We predicted that CAS would be associated with higher breast cancer risk, but we did not find such an association in our study. We also did not find a significant interaction between early life adversity and reproductive factors in shaping risk of breast cancer. Our study confirmed existing research demonstrating later AFB and low parity are significant risk factors for breast cancer.

While there was no direct relationship found between early life adversity and breast cancer diagnosis, later AFB and low parity did increase the risk of breast cancer, suggesting that reproductive factors rather than early life experience could be driving associations with female breast cancer. The role of early life adversity in shaping breast cancer risk might also be hard to identify clearly because early life adversity can accelerate the timing of first birth [29, 42, 43], protects against breast cancer risk. Together, these results demonstrate that early life adversity can have different effects on health at multiple life stages.

### CAS derived from administrative data predicts mortality

The effect of cumulative adversity is important in understanding health inequalities in adulthood [44]. Our results add to the growing literature that early adversity is associated with adulthood mortality and morbidity [39, 45–50].

The CAS measure is motivated by the same theoretical framework as the ACE measures [1, 3, 51, 52]. However, it is constructed using events recorded on multigenerational administrative records, such as parents SES status and family death records. CAS does not suffer from recall bias or social desirability effects, which is likely problematic for ACE measures [52]. Furthermore, this study demonstrates that the link between early adversity and adulthood can be meaningfully characterized using administrative records instead of retrospective surveys. Unlike most studies evaluating early life harshness, such as ACE, CAS measures structural factors of adversity, such as parental income and parental/sibling death. This is compared to more experiential measures in ACE studies, such as parental abuse and neglect, which we recognize as important measures of childhood stress provided that they can be well measured. Previous studies have shown that SES alone is a main predictor of adverse early life events occurring during childhood [53], demonstrating that growing up poor has major effects on adulthood health and well-being. Consistent with these other studies, our findings suggest that early childhood structural inequalities impact different stages of an individual’s life, including accelerate reproductive schedules and long-term health.

### Breast cancer is a disease of reproductive mismatch

Breast cancer in industrialized, large-scale populations is often characterized as a disease of ‘reproductive mismatch’, where nulliparity, low parity, earlier age of menarche and later AFB increase the risk of breast cancer. Our findings are consistent with the reproductive mismatch hypothesis for breast cancer, adding to the substantial literature demonstrating parity and AFB have a large influence on breast cancer risk [15–20, 32].

Here, we found that women with a mid- to late-AFB (>24 years old) and low parity (less than two children) are in the highest risk category for breast cancer studied here. Because of later reproduction and lower parity compared with our human ancestors, human females living in large-scale, industrialized societies, have substantially more menstrual cycles, with estimates as high as four times as many menstrual cycles in a lifetime [54, 55]. Endogenous hormones play a significant role in breast cancer risk, most notably estrogen and progesterone, by acting to enhance cell growth. More frequent exposure to circulating hormones through menstrual cycling can explain the inverse relationship between parity and breast cancer risk. Thus, it is possible that earlier reproduction likely reduces breast cancer risk simply because it is associated with higher parity and therefore fewer menstrual cycles.

In addition to hormone exposure, the relationship between early AFB and lower breast cancer risk could be due to early terminal differentiation of mammary cells induced by that first pregnancy [56, 57]. Early terminal differentiation is predicted to lower the somatic mutation burden in mammary cells, reducing the risk of cancer driver mutations that then leads to malignancy. These proposed mechanisms are not mutually exclusive: both lower cycling estrogen and early terminal differentiation can reduce the risk of breast cancer.

### Early adversity indirectly affects breast cancer risk through reproductive scheduling

We did not find a direct association between adverse childhood events and risk of developing breast cancer, but we did find that greater childhood adversity was associated with earlier reproduction and higher mortality. We conclude that these results are consistent with both a life history framework [11, 13, 27] and the transition to adulthood framework [10]. Individuals who have early childhood adversity may experience diminished parental supervision and guidance, which accelerates the transition to adulthood earlier [58]. These are similar predictions to a life history framework. Our data support the hypothesis that adverse childhood events hasten AFB, which may explain the higher overall mortality in these individuals. However, this acceleration in reproductive timing also appears to indirectly protect against breast cancer. Our results might provide insight into why some studies fail to replicate links between breast cancer and life events [25], whereas early adversity accelerates reproductive timing which indirectly reduces breast cancer risk via early initiation of fertility. Overall, we confirm longstanding findings that reproductive history has powerful effects on breast cancer risk.

### Both life history theory and evolutionary mismatch theory are necessary to understand breast cancer risk

While previous studies have found that stressful early life events are associated with an increased cancer risk later in life [1, 3, 21] for several types of cancers, our data does not support these findings. Why did we not find a relationship between early adversity and breast cancer in our current study? One possibility is that different subtypes of breast cancer are differently affected by exposure to early life adversity. An important distinction between breast cancer subtypes is hormonal receptor status, such as estrogen receptor (ER) positive or negative. Molecular phenotypes, such as hormone status, are an important factor to consider in cancer studies addressing the role of early life experience and reproductive history. Racial and ethnic minority membership are associated with a higher risk for ER-negative breast cancer [59]. Additionally, ER-negative tumors are more common among women of lower SES [60].

Currently, there is a gap in knowledge about why breast cancer subtypes have different risk profiles. Reproductive patterns have little influence on ER-negative breast cancer risk [20]. ER-negative tumors are usually diagnosed at a younger age and have higher mortality [60]. It may be that the reproductive mismatch hypothesis explains ER-positive breast cancer risk, while life history explanations and early adversity could be the underlying driver of ER-negative breast cancer risk. Further work to understand breast cancer epidemiological patterns may need to include elements of social structure and disparities combined with reproductive history, allowing us to better understand the role of social inequalities in breast cancer susceptibility.

## CONCLUSION

Our findings contribute to the growing literature on adverse early life conditions and their association with poor health and survival in adulthood. Our work demonstrates that data from administrative records, which reflect familial and social structural circumstances, such as low SES and parental or sibling death, can produce robust measures to predict the risk of early mortality. We find that early life adversity is not associated with breast cancer risk while reproductive factors (delayed reproduction and low parity) are associated with breast cancer diagnosis later life. This suggests that life history theory does not on its own explain breast cancer risk patterns and that evolutionary mismatch explanations (i.e. humans living in large-scale industrial societies having different reproductive patterns than our ancestors) should be considered as well. To the extent that early life adversity does affect breast cancer risk, it may be that this is mediated through reproductive factors, such as an association with early reproduction and higher parity, which then indirectly provide protective benefits against the disease. Future work can help disentangle the roles of these various factors by investigating the underlying molecular mechanisms and taking into account the heterogeneity of breast cancer types.

## SUPPLEMENTARY DATA

Supplementary data is available at *EMPH* online.

## Supplementary Material

eoac034_Supplementary_Data

## Data Availability

The data that support the findings of this study are available on request from the corresponding author. The data are not publicly available due to privacy or ethical restrictions.
