# Supplementary material for: Early life adversity, reproductive history and breast cancer risk
Source: Evol Med Public Health. 2022 Aug 23;10(1):429–38. doi: 10.1093/emph/eoac034 (PMC9464099; doi:10.1093/emph/eoac034)

### Supplemental Methods, Figures and Tables:

Supplement Methods:

SES Measures: Socioeconomic Index (SEI) and Nam Powers

Duncan (1961) developed the Socioeconomic Index (SEI) of All Occupations by applying census indicators of education and income for the full range of occupations to the National Opinion Research Center survey data. Using multiple regression statistical procedures, he predicted the percentage of occupations that would have favorable prestige ratings on the NORC survey based on the census socioeconomic indicators. Changes were subsequently made to allow for shifts in census occupational classification and other time-ordered variations in the data (Hauser & Warren 1997)

Nam and colleagues (Nam and Terrie 1982; Nam and Boyd (2004) developed the Nam-Powers SES scores which are based on reported occupation. Nam-Powers SES scores have the advantage of being derived in such a way that considers the association between an occupation and standard levels of education and income that are associated with that occupation (Nam and Boyd 2004). These scores are, therefore, broad indicators of SES and represent the concept more generally than discrete measures of education, income, or occupation.

Supplemental Methods Citations:

Duncan, O.D. (1961). A socioeconomic index for all occupations, pp. 109–138, in: A.J. Reiss, Jr. (ed.), Occupations and social status. New York, NY: Free Press.

Hauser, R.M. & Warren, J.R. (1997). Socioeconomic indexes for occupations: a review, update, and critique, Sociological Methodology 27(1): 177–298.

Nam, C. B., & Terrie, E. W. (1982). Measurement of socioeconomic status from United States census data. In M. G. Powers (Ed.), Measures of socioeconomic status: Current issues (pp. 29 –42). Boulder, CO : Westview Press.

Nam CB, Boyd M. Occupational status in 2000; over a century of census-based measurement. Population Research and Policy Review. 2004 Aug;23(4):327-58.

**Figure S1:** **Summary of Cumulative Adversity Score (CAS)**. CAS was developed using six demographic measures (described in methods of the main manuscript). We assigned a point for each adverse event. CAS can range from 0 to 6, however no individual in the dataset received a score of 6 adverse events. Distribution of CAS in the dataset is depicted below, with the percent of total individuals that scored a 0-5 on the CAS measures in breast cancer cases (coded 1, color = orange) and age-matched controls (coded 0, color = green).

**Model Summary**

Supplemental Methods for GLM models

To estimate the relationship between breast cancer diagnosis, reproductive history and CAS, we ran multiple generalized linear models in R. All models include breast cancer diagnosis as the outcome. The CAS model includes CAS only as the covariate, the AFB model includes the covariates of CAS and AFB, and the Parity model includes the covariates of CAS and Parity. We used separate models for AFB and Parity to isolate the effects of each in recognition of their strong collinearity. Age at first birth was classified as: 1) nulliparous 2) early = less than 20 years 3) between 20-24 years (which serves as the reference group in the statistical analyses) 4) middle = between 24-29 years and 5) late = greater than 30 years. We coded parity into comparable bins: 1) nulliparous, 2) low parity = less than or equal to 2 children, 3) between 2 and 5 children (which serves as the reference group in the statistical analyses) and 4) high parity = greater than 5 children. All models were adjusted for birth year and number of siblings.

**Table S1**. Test of linearity of CAS

To test whether the effects CAS (cumulative adversity score) has not violated assumption of linearity, we have added CAS squared term to the breast cancer models (along with the main effect of CAS) in the GLM analyses. Specifically, CAS has been mean-centered and the squared terms is the mean-centered CAS^2^. We ran the three breast cancer models with both the main effect (CAS) and main effect squared (CAS^2^). We summarized the models in the table below and report no evidence for the coefficient of the squared term being significant. This suggests we are not in violation of linearity. Summary tables generated using the stargazer package in R. The table reports the coefficient estimates (standard error), t-value of the GLM model. CAS = Cumulative Adversity Score. AFB = age at first birth.

|  | | | | | | |  |  |
| --- | --- | --- | --- | --- | --- | --- | --- | --- |
|  | | | Models | | | | |  |
|  | | |  | | | | |  |
|  | | |  | | | | |  |
|  | CAS | | | CAS + AFB | CAS + Parity |  |  |  |
|  | | | | | |  |  |  |
| CAS Squared | 0.017 (0.009) | | | 0.014 (0.009) | 0.016 (0.009) |  |  |  |
|  | t = 1.843 | | | t = 1.571 | t = 1.748 |  |  |  |
| CAS | -0.042 (0.019) | | | -0.030 (0.019) | -0.040 (0.019) |  |  |  |
|  | t = -2.153^*^ | | | t = -1.529 | t = -2.069^*^ |  |  |  |
| Nulliparous |  | | | 0.226 (0.027) |  |  |  |  |
|  |  | | | t = 8.275^***^ |  |  |  |  |
| Early AFB |  | | | -0.095 (0.023) |  |  |  |  |
|  |  | | | t = -4.060^***^ |  |  |  |  |
| Middle AFB |  | | | 0.221 (0.027) |  |  |  |  |
|  |  | | | t = 8.174^***^ |  |  |  |  |
| Late AFB |  | | | 0.249 (0.041) |  |  |  |  |
|  |  | | | t = 6.076^***^ |  |  |  |  |
| Nulliparous |  | | |  | 0.199 (0.026) |  |  |  |
|  |  | | |  | t = 7.735^***^ |  |  |  |
| Low Parity |  | | |  | 0.103 (0.023) |  |  |  |
|  |  | | |  | t = 4.512^***^ |  |  |  |
| High Parity |  | | |  | -0.203 (0.029) |  |  |  |
|  |  | | |  | t = -7.075^***^ |  |  |  |
|  | | | | | |  |  |  |
| Observations | 86,881 | | | 86,881 | 86,881 |  |  |  |
| Log Likelihood | -39,696.540 | | | -39,579.870 | -39,615.250 |  |  |  |
| Akaike Inf. Crit. | 79,403.090 | | | 79,177.730 | 79,246.500 |  |  |  |
|  | | | | | |  |  |  |
| Note: | | *p<0.05 | | | | | | |
|  | | **p<0.01 | | | | | | |
|  | | ***p<0.001 | | | | | | |

**Figure S2**: **Schoenfeld residuals test**

To test the proportional hazard assumptions of our covariate, CAS, we have plotted Schoenfeld residuals over time. We observe no pattern in the residuals, which suggests no violation of proportional hazard assumptions. Y-axis is the beta (t) for the mean centered CAS. CAS = Cumulative Adversity Score.


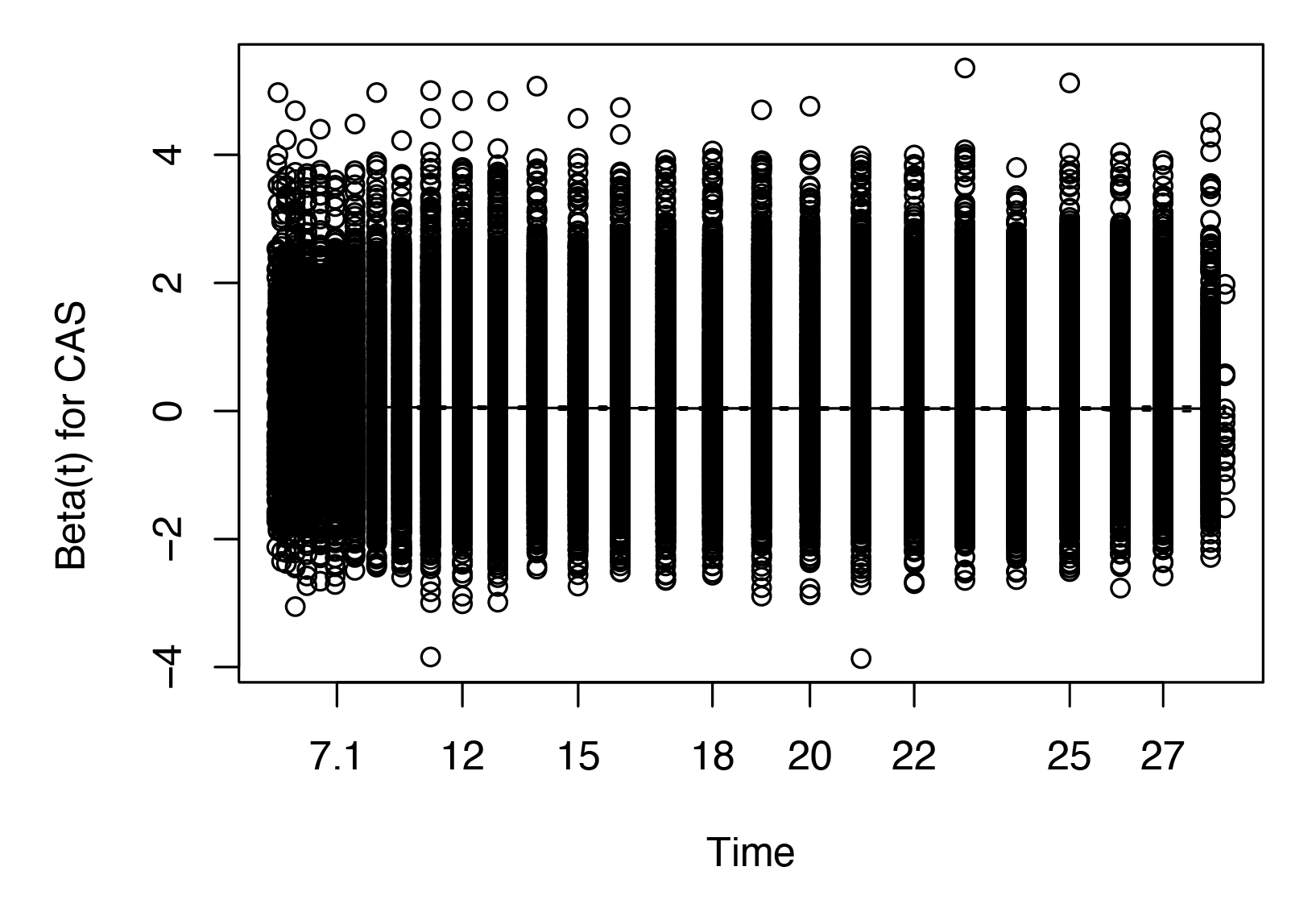


**Table S2 . Cox proportional hazards models to estimate association between CAS and all-cause mortality.** CAS significantly predicts all-cause mortality. In this model, we controlled for age at 1990 and number of siblings as covariates. Summary tables generated using the stargazer package in R. Table reports the coefficient estimates (standard error), t-value of the GLM model. CAS = Cumulative Adversity Score

|  | |
| --- | --- |
|  | Models |
|  |  |
|  |  |
|  | Cox Model 1 |
|  | |
| CAS | 0.045 (0.006) |
|  | t = 7.996^***^ |
|  | |
| Observations | 86,881 |
| R^2^ | 0.466 |
| Max. Possible R^2^ | 1.000 |
| Log Likelihood | -403,775.700 |
| Wald Test | 42,117.350^***^ (df = 3) |
| LR Test | 54,535.610^***^ (df = 3) |
| Score (Logrank) Test | 49,204.560^***^ (df = 3) |
|  | |
| *Note:* | *p<0.05 |
|  | **p<0.01 |
|  | ***p<0.001 |

**Table S3**: Summary of the relationship between CAS and reproductive variables, AFB and Parity

CAS and reproductive variables, age at first birth and parity. Controlling for birth year and number of siblings. Summary tables generated using the stargazer package in R. Table reports coefficient estimates (standard error) of the GLM model. AFB = Age at first birth. CAS = Cumulative Adversity Score

|  | | |
| --- | --- | --- |
|  | Models | |
|  |  | |
|  |  |  |
|  | AFB | Parity |
|  | | |
| CAS | -0.289^***^ (0.019) | -0.012 (0.008) |
|  | | |
| Observations | 73,801 | 73,945 |
| Log Likelihood | -213,266.700 | -153,524.300 |
| Akaike Inf. Crit. | 426,541.300 | 307,056.700 |
|  | | |
| *Note:* | ^*^p^**^p^***^p<0.01 | |

**Table S4:** Summary table of reproductive history on breast cancer diagnosis – full dataset.

To estimate the relationship between breast cancer diagnosis and reproductive history, we ran multiple generalized linear models in R. All models include breast cancer diagnosis as the outcome. These models use the full dataset (including both parous and nulliparous women). Summary tables generated using the stargazer package in R. Table reports coefficient estimates (standard error), t-value of the GLM model. AFB = Age first birth.

|  | | | |
| --- | --- | --- | --- |
|  | Models | | |
|  |  | | |
|  |  | | |
|  | AFB | Parity | |
|  | | | |
| Nulliparous | 0.240 (0.027) | 0.215 (0.026) | |
|  | t = 8.780^***^ | t = 8.383^***^ | |
| Early AFB | -0.105 (0.023) |  | |
|  | t = -4.504^***^ |  | |
| Middle AFB | 0.227 (0.027) |  | |
|  | t = 8.398^***^ |  | |
| Late AFB | 0.257 (0.041) |  | |
|  | t = 6.256^***^ |  | |
| Low Parity |  | 0.112 (0.023) | |
|  |  | t = 4.898^***^ | |
| High Parity |  | -0.215 (0.029) | |
|  |  | t = -7.530^***^ | |
|  | | | |
| Observations | 86,881 | 86,881 | |
| Log Likelihood | -39,612.920 | -39,649.780 | |
| Akaike Inf. Crit. | 79,237.840 | 79,309.550 | |
|  | | | |
| *Note:* | *p<0.05 | | |
| **p<0.01 | | |  |
| ***p<0.001 | | |  |

**Figure S3:** Effects of reproductive history on breast cancer diagnosis (**A**) Females with high parity (2 or more children) had a decreased risk of developing breast cancer compared to nulliparous or low parity (less than 2 children) individuals and (**B**) Females with an early age at first birth (less than 20 years) had a decrease risk of developing breast cancer compared to nulliparous females and females who gave birth to first child over the age of 24. All generalized linear models controlled for birth year and odds ratio were estimated from jtools [51] in R [49].


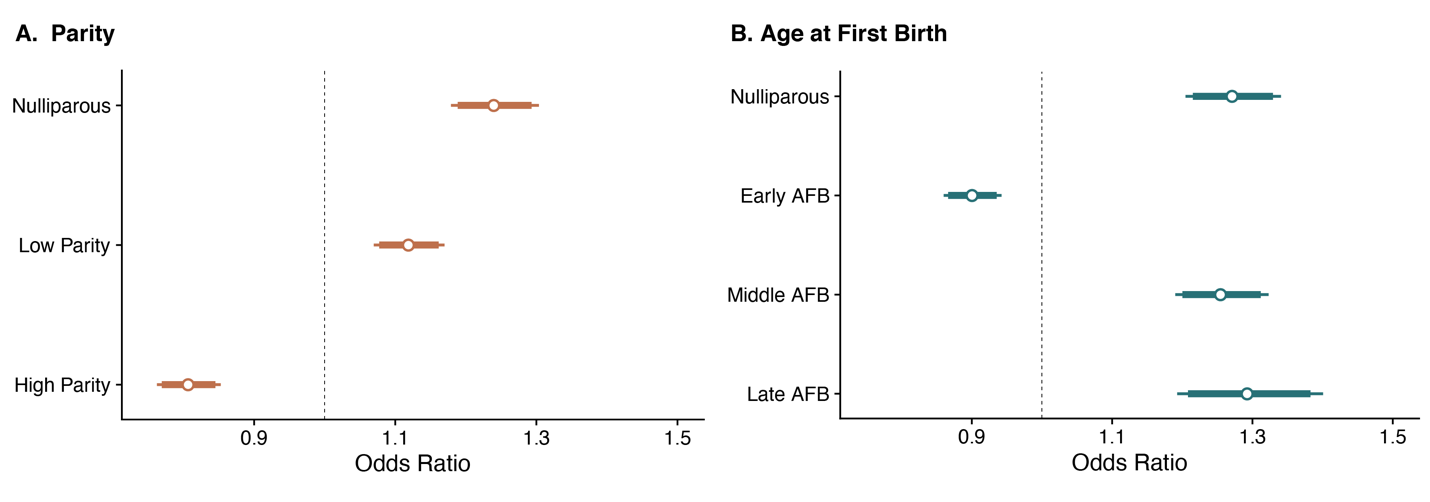


**Table S5:** Summary table of reproductive history on breast cancer diagnosis – parous dataset.

To estimate the relationship between breast cancer diagnosis and reproductive history, we ran multiple generalized linear models in R. All models include breast cancer diagnosis as the outcome. These are the summary results of the models using parous only dataset (all women had to give birth to each one child to be included in this dataset, N = 73,727 individuals). Summary tables generated using the stargazer package in R. Table reports coefficient estimates (standard error), t-value of the GLM model. AFB = Age first birth.

|  | | |
| --- | --- | --- |
|  | Models | |
|  |  | |
|  |  | |
|  | AFB | Parity |
|  | | |
| Early AFB | -0.104 (0.023) |  |
|  | t = -4.450^***^ |  |
| Middle AFB | 0.229 (0.027) |  |
|  | t = 8.472^***^ |  |
| Late AFB | 0.258 (0.041) |  |
|  | t = 6.296^***^ |  |
| Low Parity |  | 0.113 (0.023) |
|  |  | t = 4.936^***^ |
| High Parity |  | -0.217 (0.029) |
|  |  | t = -7.590^***^ |
|  | | |
| Observations | 73,727 | 73,727 |
| Log Likelihood | -33,060.600 | -33,097.830 |
| Akaike Inf. Crit. | 66,131.210 | 66,203.660 |
|  | | |
| *Note:* |  | |

*p<0.05

| **p<0.01 |
| --- |
| ***p<0.001 |

**Table S6:** Summary table of reproductive history and CAS on breast cancer diagnosis – full dataset.

To estimate the relationship between breast cancer diagnosis, CAS and reproductive history, we ran multiple generalized linear models in R. All models include breast cancer diagnosis as the outcome. These models use the full dataset (including both parous and nulliparous women). Summary tables generated using the stargazer package in R. Table reports coefficient estimates (standard error), t-value of the GLM model. CAS = Cumulative Adversity Score (mean centered). AFB = age at first birth.

|  | | | |
| --- | --- | --- | --- |
|  | Models | | |
|  |  | | |
|  |  | | |
|  | CAS | CAS + AFB | CAS + Parity |
|  | | | |
| CAS | -0.012 (0.011) | -0.005 (0.011) | -0.012 (0.011) |
|  | t = -1.128 | t = -0.424 | t = -1.118 |
| Nulliparous |  | 0.227 (0.027) | 0.199 (0.026) |
|  |  | t = 8.288^***^ | t = 7.753^***^ |
| Early AFB |  | -0.095 (0.023) |  |
|  |  | t = -4.076^***^ |  |
| Middle AFB |  | 0.221 (0.027) |  |
|  |  | t = 8.182^***^ |  |
| Late AFB |  | 0.250 (0.041) |  |
|  |  | t = 6.082^***^ |  |
| Low Parity |  |  | 0.103 (0.023) |
|  |  |  | t = 4.516^***^ |
| High Parity |  |  | -0.203 (0.029) |
|  |  |  | t = -7.074^***^ |
|  | | | |
| Observations | 86,881 | 86,881 | 86,881 |
| Log Likelihood | -39,698.230 | -39,581.090 | -39,616.770 |
| Akaike Inf. Crit. | 79,404.460 | 79,178.190 | 79,247.530 |
|  | | | |
| *Note:* | *p<0.05 | | |
|  | **p<0.01 | | |
|  | ***p<0.001 | | |

**Table S7:** Summary table of reproductive history and CAS on breast cancer diagnosis – Parous dataset.

To estimate the relationship between breast cancer diagnosis, CAS and reproductive history, we ran multiple generalized linear models in R. All models include breast cancer diagnosis as the outcome. These are the summary results of the models using parous only dataset. Summary tables generated using the stargazer package in R. Table reports coefficient estimates (standard error), t-value of the GLM model. CAS = Cumulative Adversity Score. AFB = age at first birth.

|  | | | | |  |
| --- | --- | --- | --- | --- | --- |
|  | | Models | | | |
|  | |  | | | |
|  | |  | | | |
|  | CAS | | CAS + AFB | CAS + Parity |  |
|  | | | | |  |
| CAS | -0.013 (0.012) | | -0.005 (0.012) | -0.014 (0.012) |  |
|  | t = -1.093 | | t = -0.405 | t = -1.172 |  |
| Early AFB |  | | -0.095 (0.023) |  |  |
|  |  | | t = -4.066^***^ |  |  |
| Middle AFB |  | | 0.224 (0.027) |  |  |
|  |  | | t = 8.280^***^ |  |  |
| Late AFB |  | | 0.252 (0.041) |  |  |
|  |  | | t = 6.143^***^ |  |  |
| Low Parity |  | |  | 0.106 (0.023) |  |
|  |  | |  | t = 4.601^***^ |  |
| High Parity |  | |  | -0.206 (0.029) |  |
|  |  | |  | t = -7.189^***^ |  |
|  | | | | |  |
| Observations | 73,727 | | 73,727 | 73,727 |  |
| Log Likelihood | -33,125.300 | | -33,040.240 | -33,076.250 |  |
| Akaike Inf. Crit. | 66,258.610 | | 66,094.480 | 66,164.500 |  |
|  | | | | |  |
| *Note:* | *p<0.05 | | | |  |
|  | **p<0.01 | | | |  |
|  | ***p<0.001 | | | |  |

**Table S8:** Interaction models: CAS and reproductive variables on breast cancer risk –full dataset. To test whether CAS and reproductive variables, when considered jointly, additionally influence breast cancer diagnosis beyond their main effects. These are the summary results of the models using full dataset. Summary tables generated using the stargazer package in R. Table reports coefficient estimates (standard error), t-value of the GLM model. CAS = Cumulative Adversity Score. AFB = age at first birth.

|  | | |
| --- | --- | --- |
|  | Interaction Models | |
|  |  | |
|  |  | |
|  | CAS x AFB | CAS x Parity |
|  | | |
| CAS | -0.007 (0.019) | 0.005 (0.015) |
|  | t = -0.361 | t = 0.352 |
| Nulliparous | 0.233 (0.028) | 0.211 (0.026) |
|  | t = 8.293^***^ | t = 7.962^***^ |
| Early AFB | -0.107 (0.025) |  |
|  | t = -4.313^***^ |  |
| Middle AFB | 0.226 (0.028) |  |
|  | t = 8.116^***^ |  |
| Late AFB | 0.246 (0.042) |  |
|  | t = 5.833^***^ |  |
| Low Parity |  | 0.111 (0.024) |
|  |  | t = 4.661^***^ |
| High Parity |  | -0.193 (0.030) |
|  |  | t = -6.322^***^ |
| AS x Null | -0.035 (0.032) | -0.050 (0.030) |
|  | t = -1.089 | t = -1.692 |
| AS x Early AFB | 0.029 (0.025) |  |
|  | t = 1.177 |  |
| AS x Middle AFB | -0.024 (0.031) |  |
|  | t = -0.773 |  |
| AS x Late AFB | 0.018 (0.047) |  |
|  | t = 0.383 |  |
| AS x Low Parity |  | -0.027 (0.026) |
|  |  | t = -1.058 |
| AS x High Parity |  | -0.030 (0.030) |
|  |  | t = -1.015 |
|  | | |
| Observations | 86,881 | 86,881 |
| Log Likelihood | -39,578.200 | -39,614.990 |
| Akaike Inf. Crit. | 79,180.400 | 79,249.980 |
|  | | |
| *Note:* | *p<0.05 | |
|  | **p<0.01 | |
|  | ***p<0.001 | |
|  |  | |

**Table S9:** Interaction models: CAS and reproductive variables on breast cancer risk – parous dataset. To test whether CAS and reproductive variables, when considered jointly, additionally influence breast cancer diagnosis beyond their main effects. These are the summary results of the models using the parous only dataset. Summary tables generated using the stargazer package in R. Table reports coefficient estimates (standard error), t-value of the GLM model. CAS = Cumulative Adversity Score. AFB = age at first birth.

|  | | |
| --- | --- | --- |
|  | Interaction Models | |
|  |  | |
|  |  | |
|  | CAS x AFB | CAS x Parity |
|  | | |
| CAS | -0.013 (0.019) | -0.001 (0.015) |
|  | t = -0.708 | t = -0.087 |
|  | p = 0.479 | p = 0.931 |
| Early AFB | -0.106 (0.025) |  |
|  | t = -4.271 |  |
|  | p = 0.00002^***^ |  |
| Middle AFB | 0.229 (0.028) |  |
|  | t = 8.203 |  |
|  | p = 0.000^***^ |  |
| Late AFB | 0.248 (0.042) |  |
|  | t = 5.886 |  |
|  | p = 0.000^***^ |  |
| Low Parity |  | 0.113 (0.024) |
|  |  | t = 4.754 |
|  |  | p = 0.00001^***^ |
| High Parity |  | -0.196 (0.031) |
|  |  | t = -6.418 |
|  |  | p = 0.000^***^ |
| Birth Year | 0.0001 (0.001) | -0.001 (0.001) |
|  | t = 0.127 | t = -1.016 |
|  | p = 0.899 | p = 0.310 |
| Number Sibs | -0.023 (0.004) | -0.022 (0.004) |
|  | t = -5.680 | t = -5.493 |
|  | p = 0.000^***^ | p = 0.00000^***^ |
| AS x Early AFB | 0.030 (0.025) |  |
|  | t = 1.191 |  |
|  | p = 0.234 |  |
| AS x Middle AFB | -0.024 (0.031) |  |
|  | t = -0.780 |  |
|  | p = 0.436 |  |
| AS x Late AFB | 0.017 (0.047) |  |
|  | t = 0.370 |  |
|  | p = 0.712 |  |
| AS x Low Parity |  | -0.030 (0.026) |
|  |  | t = -1.160 |
|  |  | p = 0.247 |
| AS x High Parity |  | -0.031 (0.030) |
|  |  | t = -1.021 |
|  |  | p = 0.308 |
| Constant | -1.682 (1.310) | -0.157 (1.316) |
|  | t = -1.284 | t = -0.120 |
|  | p = 0.200 | p = 0.905 |
|  | | |
| Observations | 73,727 | 73,727 |
| Log Likelihood | -33,038.480 | -33,075.300 |
| Akaike Inf. Crit. | 66,096.960 | 66,166.590 |
|  | | |
| *Note:* | *p<0.05 | |
|  | **p<0.01 | |
|  | ***p<0.001 | |
|  |  | |

**Table S10. Summary of model using CAS score binned as high vs. low.**

We have additionally estimated the models treating CAS as a dichotomous variable, where CAS <=1 is considered low adversity and CAS >1 is high adversity. We find CAS has no relationship with breast cancer diagnosis. These are the summary results of the models using the full dataset. Summary tables generated using the stargazer package in R. Table reports coefficient estimates (standard error), t-value of the GLM model. CAS = Cumulative Adversity Score. AFB = age at first birth.

|  | | | | |
| --- | --- | --- | --- | --- |
|  | Models | | |  |
|  |  | | |  |
|  |  | | |  |
|  | CASHigh | CASHigh + AFB | CASHigh + Parity |  |
|  | | | | |
| CASHigh | 0.009 (0.026) | 0.022 (0.026) | 0.009 (0.026) |  |
|  | t = 0.348 | t = 0.854 | t = 0.360 |  |
| Nulliparous |  | 0.227 (0.027) |  |  |
|  |  | t = 8.281^***^ |  |  |
| Early AFB |  | -0.097 (0.023) |  |  |
|  |  | t = -4.132^***^ |  |  |
| Middle AFB |  | 0.221 (0.027) |  |  |
|  |  | t = 8.188^***^ |  |  |
| Late AFB |  | 0.250 (0.041) |  |  |
|  |  | t = 6.088^***^ |  |  |
| Nulliparous |  |  | 0.200 (0.026) |  |
|  |  |  | t = 7.768^***^ |  |
| Low Parity |  |  | 0.103 (0.023) |  |
|  |  |  | t = 4.516^***^ |  |
| High Parity |  |  | -0.202 (0.029) |  |
|  |  |  | t = -7.065^***^ |  |
|  | | | | |
| Observations | 86,881 | 86,881 | 86,881 |  |
| Log Likelihood | -39,698.810 | -39,580.820 | -39,617.330 |  |
| Akaike Inf. Crit. | 79,405.620 | 79,177.640 | 79,248.660 |  |
|  | | | | |
| *Note:* | *p<0.05 | | |  |
|  | **p<0.01 | | |  |
|  | ***p<0.001 | | |  |

**Figure S4**. Distribution of age at breast cancer diagnosis for individuals with low childhood adversity (CAS < 1) and high childhood adversity (CAS >= 2). We observe individuals with low CAS trend towards an earlier age of diagnosis.


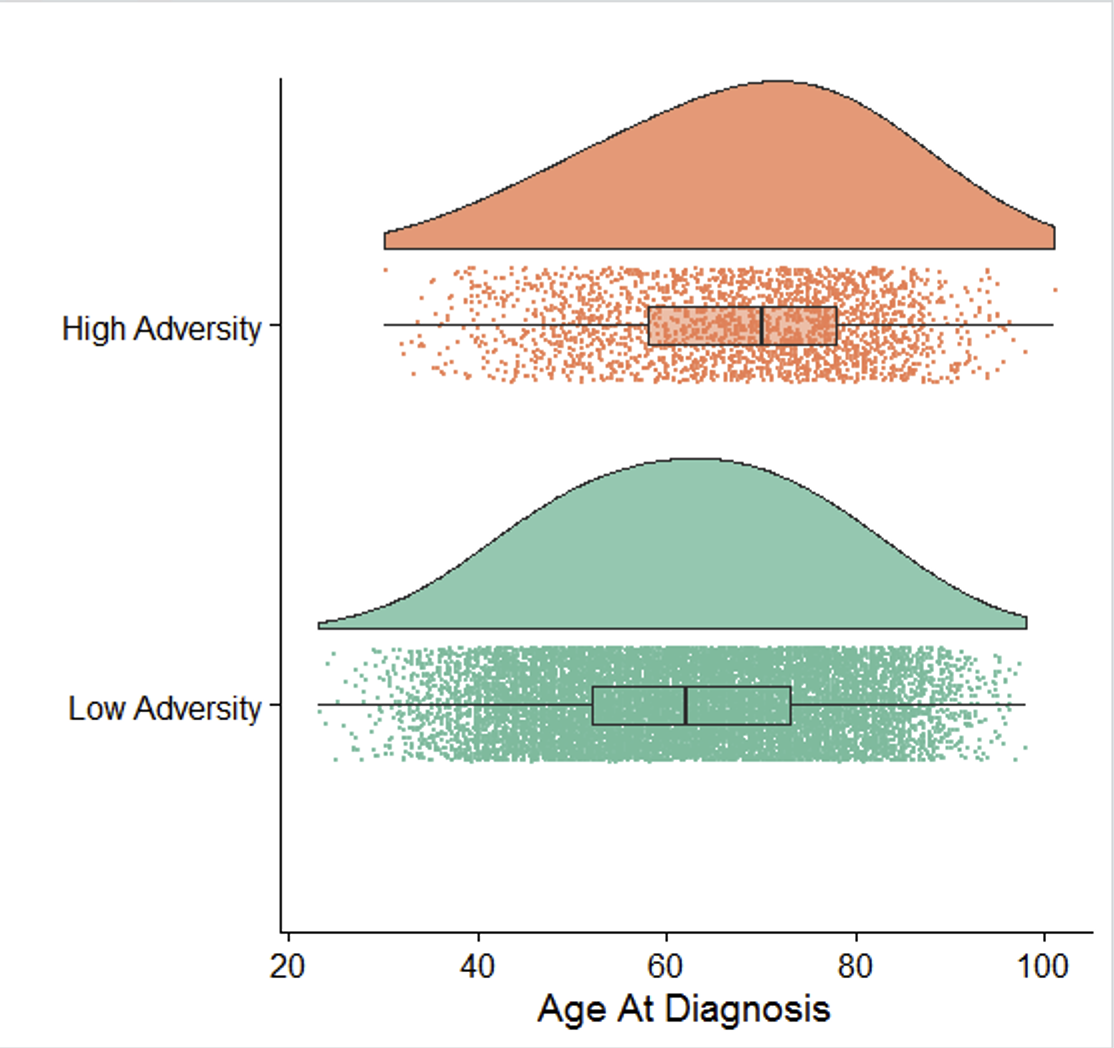

Supplement: eoac034_Supplementary_Data [file eoac034_supplementary_data.docx]
